# Data on records of indoor temperature and relative humidity in a University building

**DOI:** 10.1016/j.dib.2017.05.029

**Published:** 2017-05-18

**Authors:** O. Irulegi, A. Serra, R. Hernández

**Affiliations:** a Quality of Life in Architecture and Urbanism Group, University of the Basque Country UPV/EHU, Department of Architecture, Plaza Oñate 2, 20018 San Sebastian, Spain; b South Studio, Adarra 14, 20160 Lasarte-Oria, Spain

**Keywords:** Indoor environment, Field measurements, Educational buildings, Building retrofitting, Net zero energy buildings

## Abstract

Good indoor comfort and air quality are essential for correct educational development. Most reports in this field focus on primary and secondary school buildings, with numerous projects conducted in the Mediterranean Zone. However, little has been done in the context of university buildings. Data on indoor temperature and relative humidity data acquired trough field surveys of a seminar room located in the Architecture Faculty in San Sebastian (Spain) is provided in this paper. The seminar room was monitored during a typical spring week. The data presented in the article are related to the research article entitled Retrofit strategies towards Net Zero Energy Educational Buildings: a case study at the University of the Basque Country (Ref. 0378–7788).

**Specifications Table**

**Table:** 

Subject area	*Construction & Building technology*
More specific subject area	*Building Retrofitting Strategies towards Net Zero Energy Educational Buildings*
Type of data	*Table, text file*
How data was acquired	*Indoor temperature and Relative Humidity data were acquired trough field surveys by a wireless (SMD SHT 11) and internet-connected technology IoT gateway C4EBOX. Outdoor environmental data were acquired from the Avenida de Tolosa Weather Station of the Basque Government that is located close to the faculty building*[2]
Data format	*Raw, filtered and analyzed*
Experimental factors	
Experimental features	*A seminar room at the Architecture Faculty was monitored during a typical spring week: from 25th April to 1st May 2016. This paper presents in detail data recorded on 26*th *April and 28*th *April where maximum and minimum values were registered*
Data source location	*San Sebastian (Spain) 43°31N, 2°01W*
Data accessibility	*Data is within this article, only outdoor environmental data were acquired from the Avenida de Tolosa Weather Station of the Basque Government that is located close to the Architecture Faculty in San Sebastian*[2]*:*http://www.ingurumena.ejgv.euskadi.eus/r49-3614/es/aa17aCalidadAireWar/datohorario/contaminante?locale=es
Related research article	*Irulegi O, Ruiz-Pardo A, Serra A, Salmerón JM, Vega R. Retrofit strategies towards Net Zero Energy Educational Buildings: A case study at the University of the Basque Country. Energy and Buildings 2017 144: 387–400. (DOI:*http://dx.doi.org/10.1016/j.enbuild.2017.03.030*)*[1]

.

**Value of the data**

• The data provided in this paper might provide researchers valuable information about real indoor environmental parameters of existing educational buildings.
• The dataset in this paper might help researchers to evaluate real user comfort preferences in higher educational buildings located in a temperate climate or similar cultural context.
• The data might help researcher to evaluate the building energy performance of buildings located in a temperate climate or similar cultural context to define retrofit strategies.
• The data provided might help researcher to consider user preferences as an energy saving opportunity.

## 1 Data

The data collected in the article are related to the research article entitled *Retrofit strategies towards Net Zero Energy Educational Buildings: a case study at the University of the Basque Country (Ref. 0378–7788)* [1]. The dataset presented in this paper correspond to the indoor temperature and relative humidity of a seminar room located on the second floor of the Architecture Faculty in San Sebastian (Spain). The inputs correspond to a typical spring week: from 25th April to 1st May 2016, being the heating system off. In the following Table 1, Table 2 data for the day with the maximum values (26th April) and the day with the minimum values (28th April) are presented.

**Table 1:** Indoor and outdoor temperature and relative humidity dataset for a seminar room on 26th April 2016.

**26/04/2016**								
**Outdoor**			**Sensor 1**		**Sensor 2**		**Sensor 3**	
**TIME**	**T°**	**HR**	**T°**	**HR**	**T°**	**HR**	**T°**	**HR**
**1:00**	9.7	83	22.9	40	21.5	42	22.8	40
**2:00**	10	83	22.8	40	21.4	42	22.7	40
**3:00**	9.9	83	22.7	41	21.4	42	22.6	40
**4:00**	9.9	82	22.6	41	21.4	42	22.6	40
**5:00**	9.6	82	22.6	41	21.4	42	22.6	40
**6:00**	9.4	83	22.6	41	21.4	42	22.6	41
**7:00**	9.9	80	22.6	41	21.5	43	22.5	41
**8:00**	11.2	73	22.6	41	21.4	42	22.4	41
**9:00**	12.2	67	22.8	42	21.6	45	22.8	44
**10:00**	12.7	63	23.2	49	22	51	23.2	50
**11:00**	13.3	63	23.5	49	22.3	51	23.5	50
**12:00**	13.3	65	24.1	54	23	57	24.4	55
**13:00**	14.1	63	24	44	22.6	47	24.1	43
**14:00**	14.2	61	24.3	45	22.8	47	24.4	44
**15:00**	13.8	66	23.8	40	22.4	42	23.7	39
**16:00**	12.7	72	23.8	41	22.3	43	23.7	40
**17:00**	12.7	73	23.6	40	21.5	43	21.9	42
**18:00**	12.7	72	23.8	44	22.4	46	23.7	44
**19:00**	12.4	73	24	44	22.4	46	24.1	45
**20:00**	12.1	72	23.8	40	22.3	44	23.7	41
**21:00**	12.1	73	23.4	43	22	44	23.4	42
**22:00**	12.1	73	23.3	43	21.9	44	23.2	43
**23:00**	11.8	76	23.2	43	21.8	44	23.1	43
**0:00**	11.4	80	23.1	43	21.7	44	23	43

**Table 2:** Indoor and outdoor temperature and relative humidity dataset for a seminar room at the on 28th April 2016.

**28/04/2016**								
**Outdoor**			**Sensor 1**		**Sensor 2**		**Sensor 3**	
**TIME**	**T°**	**HR**	**T°**	**HR**	**T°**	**HR**	**T°**	**HR**
**1:00**	10.90	60	22.5	39	21.2	41	22.4	39
**2:00**	10.80	60	22.5	39	21.2	41	22.4	39
**3:00**	10.80	58	22.4	39	21.2	41	22.3	39
**4:00**	10.70	60	22.4	40	21.2	41	22.3	39
**5:00**	9.90	64	22.3	40	21.1	41	22.2	39
**6:00**	9.50	59	22.4	40	21.1	42	22.2	40
**7:00**	9.60	56	22.4	39	21.1	41	22.2	39
**8:00**	10.70	53	22.6	35	21.2	39	22.2	37
**9:00**	12.80	47	23	36	21.1	33	22.9	38
**10:00**	12.50	50	23.2	37	21.8	39	23	36
**11:00**	12.60	52	23.4	39	21.6	37	23.5	37
**12:00**	11.70	55	23.8	43	22.1	42	24.1	42
**13:00**	12.20	55	23.7	38	20	38	23.8	40
**14:00**	11.90	57	23.4	34	18.5	39	23.4	33
**15:00**	12.00	58	23.4	35	17.4	41	23.3	34
**16:00**	13.20	55	23.4	34	17.2	42	23.3	33
**17:00**	12.90	60	23.2	35	19.7	39	23.2	34
**18:00**	12.30	62	23.3	37	20.7	39	23.3	36
**19:00**	11.90	63	23.1	37	19.7	41	23	35
**20:00**	11.10	67	22.9	37	20.9	40	22.9	36
**21:00**	10.80	70	22.7	38	21	40	22.8	37
**22:00**	10.80	71	22.6	38	20.9	40	22.6	37
**23:00**	10.10	74	22.5	38	20.9	40	22.6	38
**0:00**	9.30	80	22.5	38	20.9	40	22.5	38

**Fig. 1:**
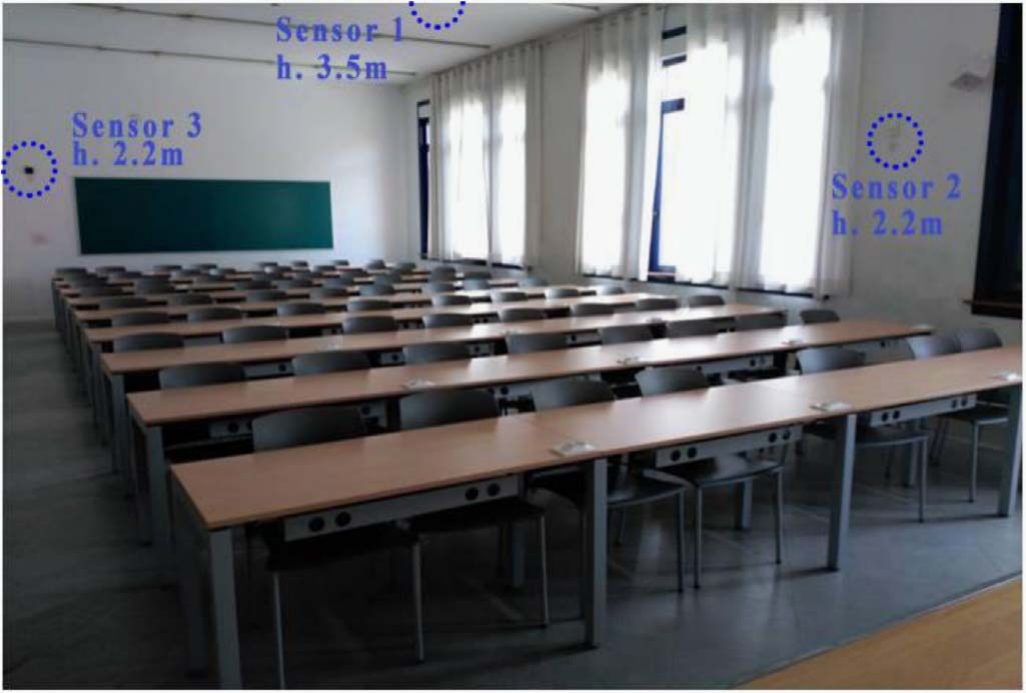
Location of sensors in a seminar room in the Architecture Faculty.

The average outdoor temperature on 26th April was 11.8 °C and the average relative humidity 73.3% (Table 1). The maximum outdoor temperature was 14.2 °C and was registered at 14:00. The minimum outdoor temperature was 9.4 °C and was registered at 06:00. The average indoor temperature on 26th April was 22.8 °C and the average relative humidity 43.55%. The maximum indoor temperature was 24.4 °C and was registered at 12:00 by the Sensor 3 (located at the back rows). The minimum indoor temperature was 21.4 °C and was registered from 02:00 to 08:00 by the Sensor 2 (located on the façade). According to occupation, 19 students were registered at 10:00 and 11 at 19:00. Location of the sensors is shown in Fig. 1.

The average outdoor temperature on 28th April was 11.29 °C and the average relative humidity 60.25% (Table 2). The maximum outdoor temperature was 13.2 °C and was registered at 16:00. The minimum outdoor temperature was 9.3 °C and was registered at midnight.

The average indoor temperature on 28th April was 22.1 °C and the average relative humidity 38.29%. The maximum indoor temperature was 24.1 °C and was registered at 12:00 by the Sensor 3 (located at the back rows). The minimum indoor temperature was 17.2 °C and was registered at 16:00 by the Sensor 2 (located on the façade). According to occupation, 21 students were registered at 09:00, 38 at 13:00 and 12:00 at 15:00. Location of the sensors is shown in Fig. 1.

## 2 Experimental design, materials and methods

### 2.1 Seminar room description

The Seminar room has a fully windowed façade facing northwest composed of 9 windows, being only the smaller ones openable. The windows are provided with blinds and curtains to control natural lighting. The lighting is provided by 5 rows of 6 fluorescents lamps and a projector connected to a PC is installed in the ceiling. The average occupancy is 20 students, usually seated in the last rows. Students usually work with their laptops. As with the rest of the building, the room is heated by radiators, positioned under the windows, with no thermostatic valves. Ventilation and air renewal is through the windows and usually takes place at break time (around 14:30) and at the end of daily classes (around 20:00). On Friday morning, classes finish at 15 h and there is no academic activity in the afternoon.

### 2.2 Data on field measurements

The Seminar room has been monitored by a wireless (SMD SHT 11) and internet-connected sensors technology IoT gateway C4EBOX where thermo-hygrometric parameters (temperature and relative humidity) are recorded every 15 min. The temperature sensors and humidity are stated respectively with an accuracy of ±1.5 °C and 3% of the measured value.

3 sensors were located as shown in Fig. 1. The monitoring campaign is to last 3 years and for security reasons, it was decided to locate sensors at a higher position than that recommended by the various standards ASHRAE 55:2010 [3] and ISO 7726:1998 [4].

Outdoor environmental data were collected from the weather station of the Basque Government, located in the Avenida Tolosa, in San Sebastian, close to the faculty building [2].

## Funding sources

The dataset is part of a research project entitled Towards a Net Zero Energy University of the Basque Country financed by the University of the Basque Country (EHUA14/20) UPV/EHU (2014 – 2016).
